# Wandering as a Sociomaterial Practice: Extending the Theorization of GPS Tracking in Cognitive Impairment

**DOI:** 10.1177/1049732318798358

**Published:** 2018-09-14

**Authors:** Joseph Wherton, Trisha Greenhalgh, Rob Procter, Sara Shaw, James Shaw

**Affiliations:** 1 University of Oxford, Oxford, United Kingdom; 2 The University of Warwick, Coventry, United Kingdom; 3 Women’s College Hospital, Toronto, Ontario, Canada

**Keywords:** wandering, cognitive impairment, social practice, structuration theory, sociotechnical practice, GPS tracking, qualitative, ethnography, action research, United Kingdom

## Abstract

Electronic tracking through global positioning systems (GPSs) is used to monitor people with cognitive impairment who “wander” outside the home. This ethnographic study explored how GPS-monitored wandering was experienced by individuals, lay carers, and professional staff. Seven in-depth case studies revealed that wandering was often an enjoyable and worthwhile activity and helped deal with uncertainty and threats to identity. In what were typically very complex care contexts, GPS devices were useful to the extent that they aligned with a wider sociomaterial care network that included lay carers, call centers, and health and social care professionals.

In this context, “safe” wandering was a collaborative accomplishment that depended on the technology’s materiality, affordances, and aesthetic properties; a distributed knowledge of the individual and the places they wandered through, and a collective and dynamic interpretation of risk. Implications for design and delivery of GPS devices and services for cognitive impairment are discussed.

## Introduction

Of the many problems faced by people with cognitive impairment and their carers, wandering is considered one of the most challenging (Cipriani, Lucetti, Nuti, & Danti, 2014; Lai & Arthur, 2003). The term “wandering,” though rarely defined, is used to describe a number of different behaviors based on the attributes of walking and movement. Attempts to understand wandering in people with cognitive impairment generally fall under two main perspectives. On one hand, it is seen as a symptom of cognitive impairment, defined according to observable actions (Martino-Saltzman, Blasch, Morris, & McNeal, 1991; Tariot, 1997). A contrasting perspective presents wandering as a *social practice*, through an appreciation of how it relates to people’s identity and sense of place (Brittain, Degnen, Gibson, Dickinson, & Robinson, 2017; Graham, 2015; Martin, Kontos, & Ward, 2013).

In this article, we draw on existing literature on wandering and technological developments for the management of wandering through the use of global positioning system (GPS) tracking technology, to propose a further shift: from viewing wandering as a social practice to a *sociomaterial practice*. We argue that it is the mutual configurability of the social and the material that is critical for successful and appropriate solutions to the challenges of wandering. We apply strong structuration theory (Greenhalgh & Stones, 2010) to analyze how GPS tracking technology is used in practice to care for people with cognitive impairment. We elaborate wandering as sociomaterial practice through detailed case studies, illustrating how wandering is (and likely to be increasingly) mediated by technology. In addition, we highlight the importance of applying a sociomaterial perspective to the development of GPS tracking solutions.

### Wandering in Cognitive Impairment

Wandering occurs frequently in people with cognitive impairment, although estimates range from 12% to 60%, due to difficulties defining and recording such instances. It is estimated that 5% of wandering instances result in the person becoming physically harmed (Petonito et al., 2013), but it often causes great anxiety to carers (Brittain et al., 2017).

Policy and clinic discourse on wandering reflects a biomedical model, defined in terms of its observed characteristics. Related terms include “eloping,” “spatial disorientation,” “agitation,” and “hyperactivity,” all have connotations of deficiency, inappropriateness, and aimlessness (Algase, Moore, Vandeweerd, & Gavin-Dreschnack, 2007; Halek & Bartholomeyczik, 2012). Biomedical research studies on wandering have focused on observable actions, such as “pacing” (repetitive back-and-forth movement), “lapping” (circling of a large area), and “random” movement (visiting several locations; Martino-Saltzman et al., 1991).

In contrast with this literature, research within the person-centered care tradition (academic nursing and critical social sciences) takes the view that identity, and sense of self resides at the level of the body and is enacted through habitual embodied actions and routines (Graham, 2015; Martin et al., 2013). In short, walking is not merely a way to travel but a *social practice*. Graham (2015), for example, uses Ingold’s (2011) concept of *wayfaring* (people inhabit the world through the embodied experience of walking) to understand the significance of movement for people with dementia living in a residential care home.

The nonbiomedical perspectives on wandering underscore the importance (and the ethical implications) of enabling freedom of movement for people with cognitive impairment. But this freedom is also associated with risks. Brittain et al. (2017), for example, found that outdoor spaces often provided positive experiences for people with cognitive impairment, but such spaces could also be threatening and unfamiliar to the person. They also found that exploring outside the home was invariably entangled with caregivers’ fears about a person’s well-being and safety. Therefore, when devising practical steps to facilitate wandering, it is important to pay particular attention to ways in which these contrasting perspectives—wandering as a healthy, meaningful practice to be supported and wandering as a dangerous and problematic practice, whose risks need to be carefully managed—may be reconciled.

The concepts of risk and risk management have become central to everyday life in late modernity (Beck, 1992). The dominant view in health care holds that risk needs to be avoided where possible, and, if not then, it must be managed within acceptable limits; who gets to set those limits and how then becomes an important issue. The field of risk assessment is founded on implicit assumptions that evaluating risk is a technical matter to be resolved through objective and rational means to minimize uncertainty. The National Service Framework for Older People in the United Kingdom, for example, talks about “risk management strategies” to reduce the risk of falling or becoming lost (Department of Health, 2001). More broadly, there have been a number of governmental risk management initiatives in health and social care, with increasing attention to ensuring standards and compliance to key areas, such as consent to treatment, personal safety, and supervision. However, such pressure can lead to a greater focus on minimizing harm to patients and avoiding more positive approaches to promoting health and social well-being that involve greater inherent risk (Taylor, 2006).

In contrast to this “objective assessment and management” approach to risk, a classic text by anthropologist Mary Douglas highlighted the ways in which hazards and dangers come to be defined by the local social and cultural context (Douglas, 1992). Risk is not part of objective reality but a multidimensional, social construct, perceived in different ways by different people in different social contexts and circumstances. This *sociocultural* approach to risk perception is important when exploring the management of wandering behavior and considering how to maximize freedom of movement for the individual, while helping maintain safety. Take the example of Atul Gawande’s moving account of the decline and death of his father (including a review of the literature), in which the tension between autonomy (the father’s priority) and safety (the priority of both his children and care professionals) loomed large (Gawande, 2014). Gawande describes this tension as one of the most important ethical conundrums of our age and makes a cogent case for careful, individualized trade-offs between support for autonomy and protection from harm. Containing and constraining the vulnerable older person on the grounds of “safety” to the exclusion of their dignity, personhood, and fulfillment, especially in the face of loss of mental capacity, is dehumanizing. A contemporary ethics of care can and must rise above such approaches. As the delivery of care increasingly relies upon technology-based interventions, the ethics of care must be woven into both its social and material practices.

In the case of wandering in the cognitively impaired, mobile devices, in particular, have assumed a growing role. Hence, understanding how these technologies are used (or why they are not used to their full potential) to support meaningful and fulfilling walking by the individual with cognitive impairment behoves us to reframe wandering: No longer can it be understood simply as a *social* practice. Instead, it has become a *sociomaterial* practice, in which the material properties and affordances of the technology and the negotiation of the relationship between these devices and the social context become key elements of the analysis.

### Using GPS Tracking to Manage the Risks of Wandering

A potential technological support for those who wander involves the person wearing a GPS tracking device (e.g., on a wristband or belt) that alerts relevant caregivers (often a remote monitoring center in the first instance, who in turn contact a nominated carer) when the device leaves a predefined geographical area (a “safe zone” bounded by a “geofence”). The use of GPS tracking to locate people with cognitive impairment is ethically controversial and divides opinion (Landau, Auslander, Werner, Shoval, & Heinik, 2010; Robinson et al., 2007). There is a perceived need in some circles to work toward greater consensus on ethical principles on who should be offered such devices and when, with calls to develop clear policies and strict procedures to protect against the “misuse” of GPS tracking (Landau & Werner, 2012; Rialle, Ollivet, Guigui, & Hervé, 2008; Welsh, Hassiotis, O’Mahoney, & Deahl, 2003). Although such efforts are laudable, it is arguable whether any attempt to resolve ethical tensions through rational assessment criteria and standardized procedures, underpinned by a set of agreed ethical principles, could possibly succeed, given that the tensions between autonomy and safety will play out differently for different individuals in different situations. The philosophical question of whether a situated, narrative approach to the ethics of GPS tracking may be more appropriate than a focus on universal principles is beyond the scope of this article (but see Pols, 2010). That aside, we argue that the balance between autonomy and safety is more likely to be achieved as a situated accomplishment, justified by a narrative account of the person-in-context, than via the technocratic application of generic principles or criteria.

Little if any research on the use of GPS devices has centered on the in-depth study of the actual experience and uses of the technology. But as we and others have previously shown more generally in relation to assisted living technologies, it is necessary to understand how such technologies are actually used “in the wild” and how people come to obtain meaning and function through their use (Gibson, Dickinson, Brittain, & Robinson, 2015; Greenhalgh et al., 2015; Greenhalgh et al., 2013; Pols, 2010; Procter et al., 2014; Roberts Mort, & Milligan, 2012; Wherton, Sugarhood, Procter, Hinder, & Greenhalgh, 2015).

The current literature focusing on the application of GPS tracking to address problems of wandering has ignored the sociomaterial dimension. Studying the interplay between the technology (and its material properties) and social agency will require research strategies to understand how the technology can shape—and become shaped by—the social roles, relationships, and perceptions in relation to the management of wandering.

### A Case Study of GPS Tracking in Cognitive Impairment

The analysis presented in this article is based on an ethnographic study of the lived experience of wandering by people with cognitive impairment and how this was managed in practice through lay and professional care networks using GPS tracking technology. The study, funded mainly by the National Institute for Health Research (NIHR), was linked to a wider program of research—Studies in Co-creating Assisted Living Solutions (SCALS) funded by the Wellcome Trust Society and Ethics Program. The SCALS program is following six organizational case studies of technology-supported health and social care, described in detail elsewhere (Greenhalgh et al., 2016). Each of these case studies involves a health or social care organization that seeks to improve care through the use of technologies; it includes an ethnographic component of the patient/client experience as well as action research with the organization to support delivery of the technological (or sociotechnical) solution.

The GPS tracking case study was conducted in partnership with the Inner City Borough (ICB) Adult Social Care service, which provides assisted living equipment and technology to people in a London borough. ICB Adult Social Care initially provided two GPS devices, which later increased to six different devices (from five technology providers) during the study. All devices included GPS tracking functionality, with tracking of the location of the user and capability of raising an alert when the wearer exits a predefined safe zone. The alerts were raised by a monitoring center operator, and the carer could also view the location using a digital map on an online portal. Beyond the GPS tracking and alert features, the devices varied with regard to functionality, design, and other material properties (see Table 1 for a summary of devices). Working with ICB and the local dementia care team, we explored the lived experience of GPS tracking technology users, their caregivers, and support service staff. We were particularly interested in their experiences with using (or choosing not to use) the GPS devices provided and the technology-supported opportunities offered by the service to better meet their needs.

**Table 1.** GPS Tracking Options Available to Service Users During the Study.

Device	Description
Buddi^TM^	GPS tracking and geofence functionality, fall detection, velocity sensor (e.g., detects movement in vehicle), and SOS button. Alerts raised through 24/7 monitoring center, with digital map access for carers. Device provided with a lanyard. Battery power lasts approx. 48 hr. During the study, company decided to stop production of this model and update to the new model (Buddi Clip^TM^, below).
Buddi Clip^TM^	GPS tracking device with geofence functionality and SOS button and two-way audio communication. Designed to clip onto clothing or lanyard. Includes additional wristband with falls detection. Alerts raised through 24/7 monitoring center, with digital map access for carers. Battery lasts approx. 48 hr and charged on central hub. The charging hub includes radio frequency beacon design to detect when the device is in the home.
Vega GPS Watch^TM^	GPS tracking device with geofence functionality and SOS button and two-way audio communication. Alerts raised through 24/7 monitoring center, with digital map access for carers. Designed to have the appearance of a digital watch. It also has a stationary charging hub, with radio frequency detection of the device in the home. Includes portable battery to charge the device while worn on the wrist. The strap can be locked onto the wrist.
Oysta Pearl+ Mobile^TM^	A basic mobile phone, with four speed dial buttons, SOS button and GPS tracking with geofence functions, fall detection, and nonmovement alerts. Can be provided stand-alone or through a 24/7 monitoring center. Includes additional mobile phone features, including calls, messaging, clock, and reminders. Battery lasts approx. 48 hr.
Mindme^TM^	GPS tracking with geofence functionality, with alerts raised through 24/7 monitoring center, with digital map access. Option of SOS button or reduced functionality, having no SOS button. Slightly smaller and lighter than the other devices, and appearance of a key ring. Battery lasts approx. 48 hr and charged on a docking stations, with automatic emails when battery power is low.
GPS SmartSole^TM^	GPS device incorporated into a shoe insole, which can be placed in the shoe of the user. Includes GPS tracking and geofence functionality, with the option of stand-alone of 24/7 monitoring, with digital map access for carers. The insole is designed to be cut to the shoe size and inserted into the shoe. Battery lasts approx. 48 hr, insole must be removed from the shoe for charging.

*Note.* GPS = global positioning system.

In this article, we describe our methodology and report our findings on how wandering was experienced and understood by persons with cognitive impairment and their carers. Using a sociomaterial theoretical perspective informed by strong structuration theory, we then explore the networks and practices involved in managing the risks of wandering using GPS technology. The key research questions were as follows:

**Research Question 1**: How and why do people with cognitive impairment engage in and experience wandering activities?
**Research Question 2:** How do members of the formal and informal care network balance the tension between autonomy and safety, with or without the aid of technologies?
**Research Question 3:** What kind of knowledge and social relations are needed to support the effective and ethical use of GPS tracking for people who “wander”?

### Theoretical Orientations

As described in detail elsewhere (Greenhalgh et al., 2016), our theoretical approach rejects the prevailing technological determinism assumed by many policy makers and biomedical researchers (the assumption that the introduction of a technology as part of a health or care service will “cause” particular intended effects such as empowerment of the patient/client, better or safer care, improvement in health outcomes, greater efficiency, and so on). Rather, we view technologies as elements in complex, dynamic systems that are typically unstable; the behavior of these systems depends on human actions, interactions, and relationships as well as on the material properties, affordances, and symbolic meanings of the technologies. Furthermore, any sociotechnical system that delivers technology-supported care has a history (and therefore a degree of path dependency); it sits within wider social structures including regulatory and political systems, and it evolves dynamically over time. Researchers who study such systems are broadly agreed that their empirical study requires naturalistic methods, particularly ethnography, but they differ in their choice of analytic approaches.

Our preferred approach is strong structuration theory (Greenhalgh & Stones, 2010), an adaptation of Giddens’ structuration theory (Giddens, 1979) that emphasizes the networked nature of social relations and the need for rigorous and detailed empirical study of small-scale social situations (*conjunctures*). Strong structuration theory analyzes the reciprocal and dynamic relationship between social structure and human agency; it divides social structures into external (meaning-systems, prevailing moral codes, political economic realities, and so on) and internal (internalized versions of these realities that are held by individuals in the form of habitus and knowledge, studied from a subjective, phenomenological perspective). Technologies, similarly, are viewed as not only generated in and by society but also as possessing inscribed *internal* social structures (e.g., role assumptions and access controls built into software) and as both creating and constraining possibilities for human action.

In common with actor–network theory, strong structuration theory holds that an individual’s social role (or *position-practice*) depends on their position in the sociotechnical network. A telecare call center staff member, for example, only becomes a “carer” because (and to the extent that) he or she is connected to a wider network of individuals and technologies involved in the support of the individual with cognitive impairment. In what actor–network theorists call *translation*, individuals within a sociotechnical network seek to mobilize other individuals and technologies to relate to one another in particular ways, so as to produce a more or less stable arrangement to achieve an ulterior goal (e.g., in this instance, safe wandering by someone with cognitive impairment). Our analysis sought to study the relationship between the (changing) network *and* how the individuals and technologies “acted” within it.

An important aspect of strong structuration theory is the detailed study of how each individual (and each technology) fits into the network and what assumptions they make about the other people and technologies in the network. An individual’s knowledge may be incomplete or flawed (e.g., a carer may believe, wrongly, that the person with cognitive impairment finds the technology intrusive or that the technology is 100% reliable). But whether flawed or not, this knowledge is an important influence on their action. Similarly, flawed assumptions built into technology (e.g., a flashing light indicating “charging” will ensure that the user keeps it plugged in) may have unintended consequences (e.g., drawing attention to the device, leading to unplugging by the cognitively impaired individual—see example in “Findings” section).

The analysis was supported by existing literature on embodied selfhood and movement in cognitive impairment (Graham, 2015; Martin et al., 2013), human geography (Middleton, 2009), social construction of risk (Hillman, Tadd, Calnan, Calnan, Bayer & Read., 2013; Tulloch & Lupton, 2003), and sociotechnical systems (Bijker & Law, 1992; Leonardi & Barley, 2008; Williams & Edge, 1996). The latter is characterized by very distinct accounts of the relationship between technology and society, ranging from technological determinism on one hand to social constructivist on the other. Leonardi and Barley (2008) adopt a position that claims a conceptual middle ground between purely deterministic or constructivist positions—technologies are adaptable but there are limits and these then push back on practices. It is this recursive relationship between technology and practice at the micro, meso, and macro levels on which strong structuration theory aims to shed some analytical light.

## Method

### Sample and Recruitment

The sample consisted of seven participants (index cases) with complex multimorbidity (cognitive and physical impairment). Participants presented different levels of severity of cognitive impairment and different physical comorbidities; they were also diverse in terms ethnicity, family settings, and social networks. Each index case was identified by the care practitioners as clients who may benefit from the provision of GPS technology and provided with the technology as part of their usual care.

The action research component involved the first author being directly involved in supporting users and addressing problems with the technology provided. Five cases were enrolled in this phase of the study, in which the researcher worked alongside service staff to resolve issues and improve the solution in place for the client, while also generating generic insights to feed into organizational learning. To this end, the researcher met with the ICB team following home visits to discuss the types of problems faced by service users and opportunities to adapt the technology or service to address such problems. These meetings provided the ICB team with a more detailed insight into the everyday experience of service users and provided a context to identify work practices that would better meet users’ needs, as well the broader organizational challenges that would need to be addressed to routinely perform these practices.

NHS Ethics approvals were granted by the NRES Committee London—Camden and Kings Cross (15/LO/0482). All participants and at least one carer provided written consent. If there was evidence that the index case participant lacked capacity to consent, then the carer would be asked to provide consent as their personal consultee. Participant and organization names and other identifiable information have been removed to maintain confidentiality.

### Data Collection and Analysis

To investigate the use and nonuse of GPS technology using strong structuration theory, we collected small-scale, detailed ethnographic data on individual technology users (*micro*) as well as organizational level data (*meso*), and wider data on the sociocultural and policy context (*macro*). Qualitative data were collected longitudinally for each index case using semistructured and narrative interviews, observations, and “tours” of indoor and outdoor spaces that participants wanted to show the researcher. Participants and their carers were visited on up to six occasions over a period of 6 to 8 months to build a rich picture of their lives, focusing mainly on specific incidents and challenges (*conjunctures* in the language of strong structuration theory). These data were supplemented by interviews with the service staff involved in the individual’s care and relevant paper and electronic documentation (e.g., assessment forms, GPS activity data). We also undertook a detailed study of the material properties of the GPS technologies in use, focusing on the affordances and constraints that shaped how it was used and how it mediated interaction across the care network.

Ethnographic study of work practices included observations and naturalistic interviews to map the people and processes involved in providing and supporting the GPS technology. This included health and social care staff, as well as staff within collaborating organizations (technology suppliers, monitoring center operators). For the action research component, the researcher engaged in discussions with service staff to explore how problems could be addressed. This aspect of data collection focused on how staff drew on their accumulated general experience and existing knowledge (flawed or otherwise) and mobilized new sources of conjuncturally specific information and knowledge to move the problem on.

Data for each index case were drawn together using narrative synthesis to produce a case summary as described previously (Greenhalgh et al., 2013). Each narrative covered (a) the participant’s social, cultural, and historical background; (b) their experience of aging and ill health; (c) the people and technologies in their life and how these were linked in relevant networks; (d) their perspective (and caregivers interpretation) on “what mattered” about outdoor and public spaces; (e) the specific GPS technology that had been offered (and which may or may not have been in use) to support them; and (f) the problems that emerged, how these were resolved (or not) over time and any unintended consequences of the efforts to resolve them.

The case narratives were used both practically (to identify service user needs in relation to activity outside the home and the roles of technological and social support, thereby informing the action research) and also theoretically (as the raw material for theorization of the lived experience of the technology and how ethical challenges emerged and were addressed).

Our analysis sought, first, to map relevant external social structures (what Stones, 2005 calls the *strategic terrain*) and the internal structures that were embodied by individuals and inscribed in the material properties and affordances of technologies. Second, we sought to document how people (the index individual with cognitive impairment and the members of his or her care network[s]) assessed particular situations and drew on their knowledge of the situation (including their assumptions and beliefs about what was ethical in the circumstances and about what other people knew and believed) and on the functionality of technologies to take particular action(s), and what the consequences (intended and unintended) of those actions were. Finally, we sought to theorize how the actions of individuals—and whether the technology “worked” [acted] as intended—fed back in the longer term to influence wider social structures (including policy assumptions and prevailing views on the ethics of surveillance).

Our interest lay in determining whether safe wandering for people with cognitive impairment was achievable through the introduction of GPS technologies and—if so—how, and how this might explain when nominally identical technical artifacts lead to quite different outcomes. This required understanding the nature of the changes in both artifacts and social practices to support safe wandering, that is, how this shaping or coevolution of the technical and the social was explored, negotiated, and achieved, by whom, and what this meant for the practice of wandering as experienced by the participants and their carers.

The starting point of this process may be characterized by a number of social and material conditions that are constitutive of the external and internal structures. In the setting of our study, these included health and social care policies and their political economic drivers; organizational rules and practices for assessing and managing risks of wandering and supporting technologically enabled care interventions to minimize those risks; the rules and practices of telecare call centers and their operators; designers’ assumptions about participants and their requirements and how these are inscribed into the artifacts; and the habitus and lived realities of the person and their families. These structures may then recursively evolve, driven by participants’ discovery of what kinds of adaptations the technology affords (not necessarily those intended or foreseen by designers) and what kinds of reconfigured social practices, both at the organizational and personal level, are necessary and feasible to deal with the limits of the technology (and vice versa). It is this dynamic that we are particularly interested in exploring, recognizing that implementation is a key site for the study of the exercise of human agency and how this is shaped by—and shapes—the artifact and the social practices within which it is being embedded.

## Findings

### Overview of Data Set

Data collection included twenty-two ethnographic visits with seven index cases and eight lay carers (approx. 50 hr), 30 hr of ethnographic visits with organizational staff (including shadowing and meeting with occupational therapists, ICB telecare coordinators, call center operators), six staff interviews with health and social care staff, and three interviews with other stakeholders (technology supplier, two monitoring center managers), and approximately 40 pages of documents (including national and local policy on assisted living, business plans, extracts from websites, emails, correspondence with technology suppliers). The seven individual case studies, structured under the six headings listed above, were between 4 and 6 pages long.

Table 2 presents the seven cases, living and care arrangement and GPS tracking technology provided. All cases were males aged 72 to 89 years. Five participants lived in their own home (one living alone) and two lived in a formal care (group care home) setting. The participants had mild to moderate cognitive impairment and considered at risk of wandering and becoming lost outside. Three participants were diagnosed with Alzheimer’s disease, two with mixed-type dementia, one with vascular dementia, and one with Korsakoff syndrome.

**Table 2.** Overview of Index Case Sample.

	Participant 1	Participant 2	Participant 3	Participant 4	Participant 5	Participant 6	Participant 7
Ethnicity	White British	Black Caribbean	White British	Asian Pakistani	Black Caribbean	Black Caribbean	White Other
Language	English	English	English	Punjabi	English	English	Hungarian, English, Russian, German
Main diagnoses	Vascular dementia, edema, stroke, heart failure	Alzheimer’s type dementia, diabetes	Alzheimer’s type dementia, asthma, high blood pressure	Mixed-type dementia, impaired hearing; urinary tract infection	Alzheimer’s type dementia, depression, dizziness	Mixed-type dementia, diabetes, back pain	Korsakoff syndrome
Home	Terraced house, owner-occupied	Group care home	Terraced house, owner-occupied	Terraced house, owner-occupied	Terraced house, owner-occupied	Terraced house, owner-occupied	Flat, supported housing
Lives with	Alone	Two other care home residents and 1–2 carers (24 hr)	Son and daughter	Wife, son, daughter-in-law, granddaughters	Wife and son	Wife	Six supported housing residents and care assistants (24-hr carers)
GPS devices	Buddi Clip^TM^	Vega GPS Watch^TM^	Buddi^TM^	Buddi Clip^TM^	Buddi Clip^TM^	Vega GPS Watch^TM^ and Mindme^TM^	Buddi^TM^ and Oysta Pearl+ Mobile^TM^

*Note.* GPS = global positioning system.

### GPS Tracking in Its Social and Historical Context

At the time of our empirical work (2015–2017), U.K. health and social care services were severely stretched as a result of “austerity measures” in the public sector (Glasby, 2017), with tightening of resources in every sphere of social work (Fenton, 2016). There was strong pressure at national policy level for local providers to identify and implement innovations to improve efficiency of service provision. Digital technologies were viewed as one important way of achieving improved services and reduced costs; they were also widely viewed as representing progress and linked in policy discourses to economic and scientific progress for the country (Greenhalgh, Procter, Wherton, Sugarhood, & Shaw, 2012). Indeed, the assumed ability of technology in general to improve the effectiveness and efficiency of services was so pervasive that government initiatives set out to encourage “digital by default” across public services and the NHS to go “paperless” by 2018 (Cabinet Office, 2012; NHS England, 2014).

Although many people with severe cognitive impairment are cared for in institutions, mild to moderate cognitive impairment is much commoner, and such individuals usually live independently or with families (Parkin & Baker, 2016). The cost of searching for a missing person is estimated to cost the police force £2,400 per case (Greene & Pakes, 2012).

Local providers of care services were thus considering GPS tracking of the cognitively impaired in a context of falling real budgets and rising need, with the threat of high and unpredictable costs of searching if wandering clients became lost. In addition, the London Metropolitan Police were working with the Adults Social Care team to promote the use of GPS tracking to reduce cost conducting search operations. The imagined solution, certainly in the minds of administrators and managers, was one in which all or most individuals with a propensity to wander would accept a GPS tracking device that they would use this device whenever they wandered outside the home, that the device would be programmable with a suitable geofence that clearly delineated “safe” from “unsafe” territory, that the alert would be triggered reliably when the individual ventured into the latter, and that a search and rescue solution by members of the user’s care network would follow logically from the alert.

The key influences on the local policy of introducing GPS tracking in our case study site were thus the bad and worsening financial situation along with a prevailing discourse of modernism (technology as efficient, clean, rational, and reliable) and increasing bureaucratic controls on social care. Social care staff took account of these influences but were also influenced by *professional* values and ethical principles, most notably the goal of enabling people with cognitive impairment to remain living at home for longer, reducing caregiver stress, and providing clients with greater freedom outdoors.

### The Individual Cases

There was wide variation in how participants experienced cognitive decline and how this related to their mobility and engagement with outdoor and public spaces. Wandering was closely tied to changes in the person’s mental and physical capabilities, chronic health such as diabetes, edema (swollen feet), and other reoccurring illness (e.g., urinary tract infections).

During the study, six of the seven participants engaged in wandering activities outside the home, which they often sought to do alone and independently. One participant did not leave the house alone during the study (to anyone’s knowledge) because he had recently experienced a fall outside the house front entrance. He was unsteady on his feet, due to low blood pressure and edema. However, he continued to move around the house when alone, resulting in a series of falls and heightened anxiety for the family.

The wandering we observed consisted of activities inside and outside the home, including repetitive movement (e.g., visiting or walking around a particular area) and actions or gestures (e.g., manipulating, dismantling, or moving objects). Carers’ attempts to control these activities or accompany them outdoors were sometimes met with resistance and conflict.

Each case was distinct in terms of clinical, social, biographical, and geographical contexts, and wandering was experienced and managed in different ways. Of particular interest for us as researchers was how this management evolved over the course of the study. For example, at the start, the ICB team offered a choice of only two GPS devices; by the end of the study, this had expanded to six different GPS device options (from five different suppliers), which varied in design and functionality (e.g., some designed to be worn or locked on the wrist, and others on a lanyard or key ring). These all had the same GPS tracking and geofence features, but different material properties that turned out to be important in terms of their acceptability.

Four of the seven participants abandoned the GPS technology they had been provided with at some point during the study. Five cases required active involvement by the researcher to help the service identify and adapt solutions in use, to address problems that would have negatively affected the sustained and effective use of the technology.

The analysis revealed three themes related to the use of GPS tracking. The remaining part of this section describes these themes using field note extracts from the case studies.

### Wandering as a Meaningful and Worthwhile Practice

Our study design, based on in-depth and longitudinal ethnographic observation, allowed our lead researcher to develop a detailed biographical and tacit (informal and implicit) knowledge of the index case. In all seven cases, it became evident over time that engagement in wandering was a meaningful and worthwhile activity for that person (and that different individuals found different kinds of meaning and fulfillment from wandering). “What mattered” to the individual powerfully shaped the ways in which the GPS technology was used, as the following fieldwork extracts illustrate.

First, wandering was important for maintaining *habitual practices* that were linked to particular places and that reinforced the person’s identity. It involved spending much of the day moving through, and acting in, socially and culturally familiar spaces. In the extract below, the participant, who is in his late eighties with Alzheimer’s type dementia, centered his daily routine around visits to the local betting shop, supported by his son who lived with him:

[Participant’s name] tells me he’s been to the bookmakers today “I stay there till my money runs out [laughs] . . . . I bet on horses and dogs . . . As long as my money lasts.” His son later explains that he does not actually follow the races. His mild cognitive impairment means that he cannot select runners, nor does he follow the race and know if he has won any money. But he still places bets by taking the betting slip from the counter, writing “FAV” (bookies’ favourite) across the front of the slip and handing it to the cashier with his cash. His son will limit how much money he takes with him, so that he is free to use whatever cash he has in his pocket. He relies on the cashier to tell him when he has won and return the winnings. But sometimes this isn’t done. This annoys his son, but he feels it important that his father goes to the bookmakers on his own and enjoys placing bets. As his son is often at home and the bookmakers is a short walk away, he can occasionally pop into the bookmakers to check on him, or collect him when necessary.

This participant’s meaningful social practice of placing bets at the local bookmakers included interacting—in ways he had done all his adult life—with the staff. This practice was actively enabled and managed by the efforts of his son, who understood the biographical significance of this practice for his father, someone who spent much of his working life as a lorry driver, traveling independently and away from home and enjoying a bet as part of the way he relaxed when away. For his son, the value of this activity significantly outweighed any monetary losses. But, it also relied on his personal and tacit knowledge of the local betting shop, how his father would act within this space, and his capacity to coordinate his own activities alongside this. For the participant, the familiar and habitual actions and gestures were important in forming *place attachment* (Degnen, 2016), in that it is not only about being physically present in this place but is also an interactional process with social and physical aspects of the environment.

The GPS solution developed for this participant was aligned with these practices, with the geofence encompassing the home, betting shop, and local pub (which the landlord would occasionally invite him to if he saw him in the betting shop), allowing him to come and go as he pleased. In this case, wandering was depicted as a problem (to be stopped) if he breached these parameters. This occurred on a number of occasions, including an incident when he went to find another betting shop nearby (with which he was less familiar and the staff and other clients did not know him) and when he went to the post office to try to withdraw his pension money.

Second, the case studies highlighted wandering as an aesthetic practice, in which the destination was less important than the experience of moving through, and interacting with, the outdoor and public spaces. Sometimes, the places to and through which the individual walked were richly evocative of positive memories (past) and/or linked to positive dreams and plans (future). In the next extract, this participant, who is in his late 80s and has mild cognitive impairment, highlights how his wandering elicits memories of his life growing up and living in Jamaica and his dreams to return there. The GPS device was requested by his wife because he was spending long periods of time out and about but could not say where he had been:

As we walked through what he calls his “plantation” (outdoor garden space where his wife has planted fruits and vegetables), he talks in a great amount of detail about the vegetation, touching and smelling them as we pass through. He stops at the sweetcorn plant to explain, in minute detail, how it is cooked and eaten in the Jamaican way. In detail he explains, with hand gestures, how the sweetcorn is cooked on an open fire, and then mixed with crushed dried coconut: “Its heaven . . . You know that God must be a good god because wherever you go the food is different . . . We are all brothers on this earth and we will all go to heaven.” As he talks freely and energetically about the plants and his culture, you wouldn’t know he had cognitive impairment. He is knowledgeable of each plant, what they are, stage of growth and when the fruit would be ready to pick. As we continue to walk, he stops suddenly, and points up at the sky, telling me to look up, quickly. Unaware of what I am meant to be looking at, and unable to follow his direction, he puts his right arm across my shoulder, positions my head with his left hand, and points at a cloud for my eye line to follow: “Wait, it’s coming . . . where is it? . . . hang on.” We stand for some time, looking up at the sky. Then, emerging from the cloud is a plane, barely visible, flying high in the distance. When I eventually see it and understand, he laughs out loud. He says he loves looking up and watching out for planes. He spends lots of time standing or sitting outdoors, looking up at the sky, watching out for them, wondering where the people are going. He used to love travelling and dreams of going back to Jamaica one day.

This extract illustrates how this participant’s wandering in the garden links him powerfully to his early roots in Jamaica and also how being outdoors makes possible his satisfying fantasies about people traveling the world and (perhaps one day) his own return to his homeland.

Third, wandering provided purpose and occupation of time, helping satisfy a need to feel useful. The following extract describes the wanderings of a participant, who is in his late seventies and originally from Pakistan. His wandering largely consisted of searching the ground and gathering objects found along his path, an activity also observed in another case in this study:

As we approach the house to visit [participant’s name] and his family, the occupational therapist tells me that this is a particularly challenging case, as he routinely engages in “searching” activity outside the home, without paying attention to people and traffic around him. She recalls her last visit, when she was getting into the car preparing to leave and saw him walk straight out of the house and across the main road. He was looking down at the ground, saw a plastic bag on the pavement and stopped to pick it up. He continued to walk, holding the plastic bag, scanning the ground by his feet as he moved, completely fixated, as if searching for something important. Hunched right over to get as close to the ground as possible, he would occasionally stop to pick something up, inspect it and place it into the bag. [Later at the house] the participant’s granddaughter tells us that his “searching” behaviour has got worse. He brings back all sorts, and even rummages through bins along the street, taking out discarded and rotten food and bringing it back to the house.

Initially, this behavior appeared to fulfill the biomedical terms often associated with wandering: purposeless, disoriented, and risky. But over the course of the study and as a result of repeated discussions with the participant and his family, the researcher and social care staff collectively came to realize the significance of this activity and how it related to his previous occupation in the textile industry:

The occupational therapist asks the family if there is anything he can do at home. There’s nothing. He doesn’t even watch television and most of the family are at work during the day. He only likes walking. There’s a back garden, but he doesn’t go out there. The occupational therapist asks the family what his previous occupation was. His granddaughter tells us that he worked in textiles, mainly sewing buttons onto bus seats. At this point she realises that, although he gathers all sorts of things, he particularly likes finding buttons, “they are like treasure to him.” The occupational therapist turns to him: “We need to find you a job.” She comes up with an idea for the family to place buttons and other interesting materials around the garden for him to search and collect.

Through their understanding of the biographical context, the family explored how this participant could continue to do the searching activity that gave him a sense of purpose and filled his time, but do so safely in the family garden rather than out in the streets.

Finally, wandering was often characterized by a *spatial temporal rhythm*, providing continuity and structure to the person’s daily life. In some cases, carers were attuned to these patterns, which helped monitor and support their activities (e.g., expecting them to return home at particular time, finding them at their “usual haunts”). As others have previously observed (Brittain et al., 2017), spatial temporal rhythms also shaped how the carer perceived wandering as meaningful and purposeful or considered it “aimless” or hazardous when the person moved out of these areas. These understandings formed the basis of the geofence configurations and carers’ decisions about how to act. In the extract below, the granddaughter of one participant describes how the family monitor his movements and synchronize their own activities with him to help use the GPS device:

“He doesn’t go out after six o’clock. After his dinner, after he has eaten, he stays in. He doesn’t go out till eight in the morning. So at six o’clock my mum will take it [GPS device] off him, because he’s in the house. And then when he wakes up, sometimes he can go out at eight o’clock, so my mum will put it in his pocket, where he will keep it all day . . . . He goes around [the park] after breakfast. And then comes back for lunch and then out again before tea time.”

### Freedom of Movement and Social Construction of Risk

Family carers’ concerns about wandering were dominated by threats to the person’s physical safety (e.g., traffic, personal security). In six of the seven cases, GPS tracking was introduced following a significant event, including occasions when the person had gone missing for a long period and found (by family or police) in an area they would not usually go to.

The various uses and configurations of the GPS technology were shaped by the differing interpretations of the risks associated with wandering, which were socially situated and influenced by different (and often changing) knowledge, values, and beliefs among carers. One key difference in the perceptions of risk associated with wandering emerged between formal care arrangements (i.e., group care or assisted living facilities) and those living with family members. In the formal setting, the focus was on mitigating potential harm to the person and knowing where they were at all times. The extract below is from a case, in which the participant, who is in his late seventies with mild cognitive impairment, lives in a group care home. The GPS geofence was set tightly around the house, so that staff could be alerted if he left the premises and ensure he did not leave unaccompanied:

[Participant’s name] will often attempt to leave the house. The care manager has been granted official authorisations to lock the front entrance, as it is believed that he will quickly become lost outdoors and presents a lack of road traffic awareness. However, the GPS tracker is still seen to be needed, as he makes attempts to leave the house when people open the front door, and he has even managed to climb out of a ground floor window. So, the tracker does not enable him to walk within a “safe zone,” but rather to help care workers to respond if he leaves the house. The geo-fence is set tightly around the house, as there are only two carers on duty at any one time, so would need to respond quickly.

In the two formal care arrangements studied, the notions of risk were constructed in relation to what group care staff considered to be competent and responsible practices for mitigating the potential harm to the individual. As described by one care service manager in the extract below, this was underpinned by fear of litigation if the person was harmed or considered to be put at unnecessary risk, as well as the need to justify resource allocations to effectively manage the person’s wandering:

“They [the authorities] do not see what we see, 24 hours a day, top to toe, literally. The moods, and the ups and downs . . . They are only interested if something goes wrong.”

Using the lens of strong structuration theory, the strategic terrain as viewed by these paid care staff was dominated by regulatory social structures (the legal and contractual conditions associated with their professional role) and by a strong sense of professional duty, as set out in the code of ethics for social workers (British Association of Social Workers, 2014) to protect their vulnerable client from harm. These social structures (as perceived and internalized by the professionals concerned) served to both create accountabilities and limit what was (believed to be) possible for them in their professional role. From the perspective of the care worker in the case above, the GPS technology had a very specific potential—to help ensure that the participant would not leave the building unaccompanied. The *actual* technical potential of the GPS device (illustrated by how it was used by other participants in our sample) was much wider, but because of their particular position-practice, these wider choices were not open to them.

In contrast, the informal care arrangements involved a reciprocal care relationship and a sense of responsibility by families to ensure the person’s safety and material comfort while also helping them achieve fulfillment and happiness and respecting their independence. This involved a pragmatic and ongoing balancing of the tension between autonomy and safety. In the following extract, the granddaughter of one participant illustrates how the family’s relationship and biographical knowledge of them informs their judgments about how and when to control his movements following his fall after an episode where he became lost and tired while wandering:

“It came to a decision, should we lock the main doors and not let him out? But he would get really angry and upset. And we can’t do that because he really enjoys walks. It is the only thing he can do and he really enjoys it . . . . He’s always liked to walk. He never used public transport. One day we thought to lock the door. But that wouldn’t work because he’d get frustrated and angry.”

Decisions to balance autonomy with safety were not limited to concerns about the individual’s physical safety. Some carers had wider concerns, including a need to protect their own time or psychosocial resources, and opt for situations that were more manageable. Family members typically had multiple accountabilities (job commitments, other dependents) and/or limited physical strength or patience; it was simply not practically possible to be “on call” for the wandering individual 24 hr a day. Using the lens of strong structuration theory, each family member was situated in a social network; their multiple accountabilities were defined by the social expectations associated with particular kinship ties (e.g., father–daughter) or other social relationships (e.g., employer–employee). Different solutions to the challenges of the individual’s wandering would put different kinds of strain on the network of accountability.

Within the network, technologies (telephone, email, GPS tracking device) were used pragmatically and creatively to support activity by and around the index case, but—as with the formal carers—technology use was limited by how the lay carer saw the wider strategic terrain. The (perceived) *possibilities* for how the GPS device *could* be used were limited, for example, by his relatives’ culturally shaped views on how a British Asian daughter or granddaughter *should* behave toward an older male relative. In this case, the women considered it highly appropriate for them to allocate many hours per day to walking with and searching for him. Relatives of some other index cases held different perspectives, depending on their cultural background and competing accountabilities.

As others have previously found (Kindell, Sage, Wilkinson, & Keady, 2014; Oyebode, Bradley, & Allen, 2013), lay carers can also be concerned about other people’s perceptions of their relative. Our data illustrated that (unlike professional care workers), lay carers’ actions around wandering were sometimes influenced by their perceptions of how other people viewed the behavior. In the extract below, for example, one family member was concerned about how their relative’s wandering behavior might be perceived or misunderstood by members of the public:

“They [call centre operator] called, and he was on Queen Road. What I panicked about, because it was half-past eight in the morning, and he was just sitting there, on the road. It’s a school day, and that’s a girl’s school. And the parents might see him there, thinking he’s watching them. That’s not what he’s doing. He’s lost. But my worry is what they are thinking, what he is doing there. That was my concern then.”

In this example, the participant’s meaningful wandering routine takes him past a girls’ school. As he stops near to the school his behavior is open to the very different interpretation of sexual predation. In a society alert to pedophiles, the knowledge of how to (in Giddens’ terminology) “go on” in society would include an awareness among older men not to linger outside a girls’ school. The participant’s family are concerned that as he has lost this awareness because of his cognitive decline, his behavior could generate the unintended consequence of confrontation or even arrest. This example illustrates how the individual’s “freedom to wander” was, in reality, restricted by the fact that his behavior was socially inappropriate and open to misinterpretation *by people who did not know him well*.

Our data set also contained examples of neighbors and acquaintances who did know the individual well enough to interpret their behavior and take account of their cognitive impairment, thus enhancing the person’s freedom of movement. This *knowing well* included recognizing the individual, understanding where they tended to go, their familiarity with the setting, and how they were likely to act in these places. In some cases, the safe zones on the GPS device were set to local areas that the person was familiar with and also where they were known to the various people in these settings. In the extract below, the daughter of one participant describes how the wider social network (that went far beyond immediate family) could “keep an eye” and assist if needed:

“He is pretty lucky here because all the neighbours know him, mostly. And if they did see him walking [outside the estate], they would probably fetch him back. The neighbours next door, they have lived here since we’ve lived here. So, they would fetch him or hopefully someone will see him.”

Such tacit knowledge of the person and the local area played an important role in carers’ interpretation of GPS information and response to alerts. For example, in the extract below the granddaughter of one participant describes how the shared knowledge and capabilities across the family network supported their capacity to enable greater flexibility in response to geofence breaches:

“If he has gone to the corner shop, they [call operator] will say he’s out of his boundary, but we know he comes back. If he doesn’t come back within ten minutes, we will look where he is . . . About three times a week [they get a call], and twice out of that three we know where he is . . . . If they say three roads away or further, we know he’s not familiar with the area.”

### Everyday Risk Management of the GPS System

Although GPS devices were implemented to minimize risk, they also introduced new risks associated with complexity of both technical aspects (e.g., false alerts due to erroneous GPS readings) and social aspects (e.g., wearing the device, charging it in the house). Resolving such problems demanded a great degree of “tinkering” and adaptation by carers, and doing so while it was in use. For example, some carers sought to induce “covert” use of the device (e.g., by hiding it in clothing) and avoided talking about it with the participant, to minimize risk of confusion, distress, and fiddling with the device. In the extract below, the son of one participant describes his efforts to minimize his father’s awareness of the device as he attempts to charge it overnight:

“A problem I have with Dad is that when I put it on charge, normally I sit it on there [footstool in the living room]. But when Dad gets up in the night. Cus he does get up at night. He sees the flashing light there and goes over and disconnects it. So that’s a problem . . . So what I do now is push it down there behind the [footstool] so it’s not something he’ll see when he looks down.”

Everyday risk management strategies extended beyond the informal care network. Drawing on their own knowledge of the practical challenges, the ICB telecare coordinators utilized the GPS device portal (initially accessed to set up the device settings and alert parameters) to monitor the battery levels and GPS readings, to confirm that it was being adequately charged and used. If they detected that the device was not being charged regularly, then they would contact the carer to encourage use or resolve any issues. They were well aware that such calls could be intrusive and engender a sense of being under surveillance. This was further managed through their social interactions and relationship formation with carers over the phone, as explained by one of the ICB Telecare Coordinators below:

“So we just call them to say “Hi,” just be quite general. It’s not like a telling off. It’s just, “Oh, remember that Mr Smith has a GPS device and it needs to be charged every night, and we can see on the system that it’s only got 30% battery left, so you might want to put it on charge for a while". It’s just a gentle approach that we take with them. We don’t want them to think “We’re watching you.””

For the GPS monitoring center operators, strategies evolved to overcome the challenges of communicating the person’s location to the “responder” on the ground. The following extract highlights an ICB Telecare Coordinator’s account of two very different practices (and outcomes) with similar GPS technology. In one monitoring center, staff learnt to cross the boundaries of standardized protocols to inform, and work with, the responder on the ground. In the other case, interactions with the responders were structured along fixed protocols:

“We have had positive feedback from carers about [monitoring center A]. The operator doesn’t close the call until the person is found, so that makes the difference. The family is in constant contact and the operator was actually willing to call the person and say, “Okay, well this person has now moved from here to there . . .” With the other [monitoring center B], they weren’t very helpful—just said oh, he’s at [road name] and that was it. Obviously, he’s not going to stay there. There was no “Okay, I’ll stay on the phone" or "I’ll call you back in another ten minutes.” Because remember, they’ve got to go and retrieve their relative. So, by the time they get there, they could be somewhere else. And when they phone back, they had to go through the whole process again. They called the center and it was a different person who answered . . . there was no continuity.”

Another challenge was introducing and explaining the GPS device to the person and their family. In particular, support staff and carers were faced with the sensitive challenge of gaining agreement of the person to wear or carry the device, while enabling them to feel that they were maintaining their dignity and freedom of choice. In one case, an ICB team member visited the participant after he had refused to wear it on his wrist, saying his wife was tagging him “like a dog.” The ICB team member decided to meet him in person (something they rarely have time or resources to do), present an alternative option (to carry the device on a key ring), and represented it in a way that he would find more acceptable. The extract below presents the ICB team member’s experience of this encounter and how it supported continued use:

“There was a connection. We are both from the Caribbean. He wanted to take me on holiday [laughs]. He didn’t really want to talk about the GPS. We kept bringing him back onto it. He knew he had it, but couldn’t see the reason why he should take it with him. I was telling him—he called me his girl lollipop—I said he must take it on his keys. I said “You need to put it on your key and keep it on there!” [laughs] . . . . But we have had other conversations after that. Because I could see he wasn’t using it, about a month later, and I phoned the wife and said “I can’t see him using it—what’s going on?” And she goes “Oh he doesn’t want to put it on the key" . . . . So I got him on the phone. He was giving me these stories, “It’s too big, is it ok if I just keep it in my pocket.” I said “Yes! Just keep it on you.””

These interpersonal aspects of the delivery and use of GPS devices were achieved by working around the formal work processes and duties (the regulatory structures mentioned above) intended to manage and regulate risk and deliver efficient and standardized care. In many cases, it was felt that the organizational structures and protocols actually impeded their effort to help users on a personal level in relation to the management of wandering.

The tacit knowledge (know how) and hidden work (work not formally recognized or remunerated) involved in supporting the use of the technology were not the product of the service protocols or structures but of the informal and personal interactions and relationships that developed over time as the care workers got to know the clients and family members. As is strikingly illustrated by the quote “we’re both from the Caribbean” and the very informal and humor-ridden exchange that ensued, this care worker’s relationship with the service user is not *merely* a “system” (social worker–client) one but also a shared understanding or *lifeworld* one (common ethnic ties; Habermas, 1987). The latter brought different accountabilities and reciprocities, which the care worker used to help cajole him into using the device.

## Discussion

### Meaningful Wandering and Telecare Surveillance

There is a growing body of literature in the person-centered care movement that puts the body and embodied practices at the center of exploring how dementia is experienced. This has progressed understanding of cognitive impairment beyond deficit-focused accounts that characterize biomedical thinking and directed attention to the significance of embodied agency in dementia care (Kontos, 2005). This work has largely focused on long-term residential care settings, in which institutional regimes for order and efficiency (e.g., clothing, sleep/wake patterns, and mealtime routines) preclude important embodied and social aspects of the activities (Martin & Bartlett, 2007; Twigg, 2010). Kontos and Martin (2013) also warn of the biomedical assumptions underpinning the deployment of monitoring technology to help control and manage “pathological” behavior patterns in residential care.

Following previous studies on wandering as an embodied practice in cognitive impairment (Brittain et al., 2017; Graham, 2015), findings from our study of real case participants in context has highlighted the need to acknowledge wandering as a potentially meaningful and worthwhile activity. Our findings highlight first that wandering may support habitual activities linked to particular places and social settings that the person can make sense of and belong to. Actions and gestures that may be defined as “disinhibition” or “agitation” from the cognitive perspective can present meaning when considered as an embodied practice. Second, participants draw attention to the intrinsic value of esthetic, rather than purposive, walking (Wunderlich, 2008), shaping a person’s mood, thoughts, and preserving links with the past. Third, following (Feil & de Klerk-Rubin, 2002), wandering appeared to help satisfy an unmet need to feel useful, as one may have felt when engaging in family, home, and working life. The absence of meaning in a situation leads to boredom—an emotional feeling of anxiety, resulting in a restless feeling that there is need to get on with something interesting (Barbalet, 1999). As the example of the participant (an ex–textile worker) collecting buttons illustrates, actions and gestures which at face value appear aimless could be viewed as holding (at least momentarily) symbolic occupation and a productive use of time.

To date, the biomedical notion of wandering has formed the basis of GPS tracking development, including recent technical advancements applying machine learning algorithms to distinguish between “mobility trajectories,” such as pacing and lapping (Lin, Zhang, Huang, Ni, & Zhou, 2012). However, the individual case narratives highlight the potential limiting factor of GPS tracking interventions if the design and implementation is not grounded in an understanding of the ways that people experience and live with wandering. In addition, they highlight that individual use (and nonuse) of GPS tracking technology is embedded within, and dependent on, a particular network of social relationships and position-practices. Indeed, technology-supported wandering for our participants was revealed as a *sociomaterial practice*, in which the mutual configurability of the social and the technical was critical for success.

Key to the collaboration and decisions made by particular actors in the sociotechnical network was what those actors *knew* (correctly or incorrectly) about other actors, both human and technological. The social relations and accountabilities within the network and the material properties of the technologies, both created opportunities and constrained choices, making some options appear more possible and/or more or less ethical.

### Embodiment and Cognitive Impairment: Care at a Distance

The study of wandering in the community setting has illustrated that, as Beck (1992) observed, risk-taking permeates many aspects of ordinary, daily life. Studies on the “governmentality of risk” in health and social care work practices have shown how organizational structures intended to eradicate uncertainty of patient safety and standardize care provision can inadvertently erode the person-centered interactions with patients and shift focus onto the systems of risk regulation. Through her analysis of interactions between care workers and patients in acute care wards, Hillman et al. (2013) showed how governance strategies and patient safety regulations ended up impacting the caring relationships in ways that compromised patients’ autonomy and dignity. In the present study, risk governance affected formal care workers’ and managers’ priorities in relation to patient safety, underpinned by fears of legislation and amplified by their interpretation of the GPS device as an organizational risk management system (with geofence parameters and alert protocols), which they were responsible for implementing effectively to further maximize the client’s safety. Furthermore, risk governance structures affected care practitioners’ capacity to engage with users on a personal and ongoing basis as their attention turned to the administrative duties and paperwork whenever they engaged with and supported the user and family. Such restrictions are, arguably, a more or less inevitable consequence of the current regulatory and professional structures within which these workers were positioned. Our longitudinal analysis showed how, over time, some care workers were able to build informal, kinship-like relationships with clients and their families based on interpersonal ties rather than organizational roles—and to utilize these relationships to persuade clients to use the GPS technologies.

We have seen how telecare (specifically GPS tracking) in a community setting involves numerous people supporting the individual, with distributed roles, responsibilities and knowledge, and working across different organizations. In many such cases, achieving a meaningful understanding of the person’s wandering in context will be very challenging. Different people will have varying degrees of interaction with, and knowledge and/or representations of, the person and their wandering activities—and most will have little or no human contact with the individual. For example, to GPS monitoring center operators, the person is represented with minimal personal information and a coordinate on a digitized map. Despite this, they are often drawn into undertaking *emotional* labor (Procter et al., 2016), providing social contact and developing ways to support intersubjective sensemaking. Similarly, the ICB team’s initial interactions with users were framed by their distant role of setting up and configuring the GPS technology using the online portal. But over time, they devised ways to use this information to understand what was happening at home, supported by relationship formation over the phone, and in some cases by visiting service users and their carers at home. This human engagement is encouraging given Bauman’s (1989) warning of the dangers of modernist bureaucracies. He argued that through hierarchical structures, the use of technology to achieve control at a distance, and a highly formalized division of labor, humans can become detached from the reality of their work and fail to take moral responsibility for the consequences of their actions.

The sociomaterial perspective highlights how support for wandering as a meaningful practice would require greater attention to ways to support these interactions and relationships that help harness knowledge and understanding of the person and their wandering activities.

### Situated Judgments on Autonomy and Risk

Previous studies have shown how the introduction of new technology in care settings can challenge existing assumptions, values, and ways of working. Dealing with moral conflict and change is part of the fitting and tinkering of technological applications within everyday care activities (Kamphof, 2017; Pols, 2010). Kamphof (2017) draws on the notion of *reflection-in-action* (Schon, 1983) to describe the ways in which care home workers engaged with new telemonitoring technology, which challenged their ethical values on privacy and dignity of residents. Carers initially felt a need to be absolutely open to clients about observations to be respectful. But over time, they discovered that weighing what to watch or ignore, and what and how to communicate this information to residents, while keeping a relationship of trust was needed to work with the technology.

Similarly, in this study, the formal organizational roles and regulatory structures dominated the actions and perceived capabilities of the GPS solution. But over time, relationships developed with users (and across services) and the organizational structures exerted less influence, with lifeworld expectations and values presenting a more significant influence. Carers and practitioners developed ways to deal with the complex social and technical realities of everyday use of GPS devices. This included ways to talk about and represent the technology so as to not disrupt the person’s dignity, but also address issues of acceptability and continued use. In some cases, “covert” strategies were employed by carers to avoid distress or disruption to the system dependability. We also observed how the ICB team took the initiative to routinely monitor the status and movement of each individual’s device to check it was being sufficiently used and maintained, while making efforts to minimize the risk of carers feeling as though they were being watched. This complex and evolving relationship between the people and technology was necessary to deal with the uncertainty and risk that the tracking system introduced.

Evetts (2006) points out two types of professionalism in decision making, termed *organizational* *professionalism* (control lies with rational–legal forms of decision making) and *occupational professionalism* (collegial authority, drawing knowledge and values beyond formal procedures). In studying social care practices, Fenton (2016) warns of the threat that the, increasingly dominant, organizational professionalism framework presents to a working knowledge of the “right thing to do”, the ability to work with service users and put their interests first. She proposes a need for social care to become more conducive to occupation values and a sense of agency rather than procedural sources of knowledge for decision making.

This suggests that ethical debate on the use of GPS should shift from a pursuit for consensus and instead focus on the ways in which people deal with the complexities of use and how occupational professionalism can be enhanced. This will require greater attention to people’s position-practice across the sociotechnical network and how to enable capabilities to adapt and support appropriate and workable solutions. The exact nature of such adaptations, however, must remain open to continuing review to remain effective in the face of changes in the external structures—that is, social care policies and their political economic drivers—and the internal structures—that is, material affordances of the technologies—and how these shape, and are shaped by, the knowledge held within the care network.

## Conclusion

In this article, we explore how GPS tracking technology is used in practice to support people with cognitive impairment. The application of a sociomaterial perspective seen through a strong structuration lens has revealed the ways in which members of the care network dealt with the social and technical realities of using and supporting GPS tracking solutions.

The findings suggest that current research and debate on the appropriate use of GPS tracking and the ethical implications are misplaced as it has been considered in isolation from everyday use. Greater attention needs to be paid to the ways in which people deal with the social and technical complexities of use and how this can be fed back into the development of the sociotechnical infrastructure—as embodied in the external and internal structures—so that it is better able to adapt and thereby support more meaningful wandering practices more effectively.
